# The Treatment of Repressed Memories by Hypnotism

**Published:** 1926

**Authors:** Percival S. Connellan

**Affiliations:** London


### THE TREATMENT OF REPRESSED MEMORIES BY HYPNOTISM.

BY

PERCIVAL S. CONNELLAN, M.R.C.S.

(*London.*)

---

HYPNOTISM was first used in the treatment of repressed memories by Freud and Breuer. They, however, abandoned it in favour of the much more lengthy and complicated process of psycho-analysis, because the results were "capricious and not permanent." There is, however, very clear evidence that many cases can be so treated and cured; and the process is so simple that it seems a pity such a valuable aid in our treatment of a group of patients who come to us suffering from severe illness should be neglected. With the prospect of establishing a cure of a life-long disease in a few days there seems no reason why, in suitable cases, the patient should not be given the benefit of treatment by direct suggestion.

1. *Definition of Repressed Memory.*—A memory which, in childhood, is implanted upon the subconscious mind under such circumstances that it is forgotten, and which later produces symptoms of illness, mental or physical, is a repressed memory. This definition is open to many objections; but, as a basis of explanation, it suffices to show us the origin of the disease we have to treat.

It is taken for granted that everyone has repressed memories of painful incidents which, in the majority of cases, lead to no harmful results, repression being Nature's method of dealing with painful experiences. But in certain cases, because of some mental overstrain in later life, sometimes under conditions presenting associated ideas, the repressed memory acts as an irritant, and produces illness out of all proportion to the mental distress which is the immediate exciting cause. With the increased turmoil of modern civilisation these cases are becoming more common, as well as more insistent that we shall cure them, and allow them to take their place in the conflict of life.

2. *Symptoms.*—These may cover anything, from severe pain to a simple state of exhaustion, because they reproduce what occurred at the time the memory was repressed. Certain symptoms, as will be explained later, are suggestive of this nervous condition; but the only true test is the exclusion of all physical causes, or the failure of the patient to respond to the ordinary treatment of the ostensible cause.

3. *Treatment.*—As the illness is due chiefly to the repression of the memory and not to the memory itself, the object of the physician must be to recall to the patient's conscious mind the forgotten incident which is acting like an explosive energy in an enclosed space, or like a force which, coming up against an impregnable brick wall, rebounds and gathers strength for a fresh effort. Take a hypothetical case. Suppose (to take an extreme case) one saw one's father run over and killed by a taxi; in future the sight of a taxi would impose a horror which the revived memory of the accident would explain. One would reason that such a thing could not occur again, and in time would become accustomed to seeing taxis without horror. But if, as often happens in childhood, the memory of the sight is repressed, then every time one sees a taxi the horror arises. The subconscious memory rises also, but does not reach the conscious mind, rebounding, as it were, from the brick wall and increasing the horror, which remains unexplained, until one finds that one has such a horror of taxis that one cannot bear to see them at all.

It will be seen from this hypothetical case that, if we can recall the memory into the conscious mind the symptoms will be explained, and reason will adjust the recollection. The repressed memory is seldom of so serious a character as this, but is generally of an almost trifling nature, as in the case illustrated below.

In order to recall the memory only the first stage of hypnosis is usually necessary, *i.e.* that stage in which the voluntary movements most frequently accomplished by reflex action are inhibited. Ninety per cent. of people are capable of being hypnotised to this extent.

The patient is placed in a recumbent position in order that complete relaxation may be obtained. I do not begin until he is thoroughly comfortable. Then he is asked to concentrate all his attention on a particular object (usually the back of my watch), held about a foot from his eyes. After a few moments, while gently stroking his forehead I suggest that he is growing drowsy, that his eyelids are growing heavy, and tell him that he may blink. After suggesting this once or twice, if I notice the eyelid drooping, he is told to close his eyes, and then make the first inhibitory suggestion, that he cannot open them. I again suggest that he is drowsy, and tell him to sleep. The suggestion is then repeated that he cannot open his eyes. If I notice a flicker of the eyelids I repeat it more strongly, and tell him that he may try to open them but that he cannot—that he doesn't want to do so. If the eyes are definitely closed, so that he cannot open them, and the patient is comfortable and relaxed, this is all that is necessary.

It is better on the first occasion not to try to recall the memory, but to allow the patient to become accustomed to the restful sensation of hypnosis ; then, on the second or third visit, when confidence is mutually established, I proceed with the suggestion that he shall remember the incident in early childhood when his symptoms first declared themselves, and leave him. On returning after an interval of a few minutes I ask him if he can remember anything. Generally he visualises something which has no connection with the symptoms, but which will later become an important piece of a kind of mental jigsaw puzzle. After again stroking his forehead and telling him to sleep, I leave him to recall something further.

The process of recalling a repressed memory under hypnosis is rather exhausting to the patient ; it is well therefore to proceed slowly. Sometimes the whole memory comes up in one effort, but more frequently in separate pieces both during hypnosis and after, until the whole incident is pieced together, as the following case will show.

On February 14th, 1923, Miss A. B. was brought to me by her doctor with the assurance that nothing in her physical condition could be found to account for the symptoms. She gave me the following history : She suffered from attacks of weakness, which, coming on suddenly, were accompanied by great depression. The duration varied from a few days to weeks. They occurred at intervals varying from six weeks to three months. Frequently they were accompanied by a rise of temperature to 101° or 102°, and on these occasions she was generally labelled "influenza." Sometimes she had a pain in the side of her chest. She had spent six months in hospitals and sanatoria under suspicion of tuberculosis ; but the presence of this malady could never be proved. The first remembered attack occurred at the age of 14; and, with slightly varying symptoms, such as the rise of temperature, pain in the chest, cough, headache or sore throat, but always with pronounced weakness and depression, they had recurred ever since. On physical examination I could find no evidence of organic disease, except some defects of the teeth, which were remedied later. I explained briefly the nature of repressed memories and of hypnotism, and induced a mild feeling of drowsiness.

On February 16th I hypnotised her as far as the first stage, and removed a headache by suggestion. I then suggested that she should recall the cause of her distressing symptoms in some incident which took place when she was a small child. She did not remember any incident.

On February 17th she reported that on the previous evening the memory of two forgotten incidents had come to her: 1. A warm castor oil plaster was being placed on her chest. 2. She was looking downstairs and feeling afraid.

I again hypnotised her lightly, and she recalled the memory of a staircase, and seeing a large black object, which frightened her; also of crying and of her mother telling her not to be silly.

On February 19th she reported that she had remembered her mother's presence in the next room, and her distress that she did not come to her when she called.

I again hypnotised her; and this time the disjointed pieces formed themselves into this clear picture. A child, standing at the top of a staircase in her nightgown, has been sent to see what has caused a noise in the dining-room. At that moment the shadow of a head appears upon the blind of the window at the bottom of the stairs, because someone has passed on the pavement outside, intercepting the light from the street lamp. The child is frightened, and has a sensation of weakness in the legs, which refuse to move. She cries, her mother comes out of the room, and, telling her not to be silly, passes her and goes down the stairs. The child collapses, and lies for some time with the draught from a window behind blowing on her side. Then, picking herself up, she goes to her bed, where she feels very much alone and unwanted, and decides not to appeal for help again.

On February 21st she reported that the memory was now complete, certain minor additions having been recalled. She also said that she was sure she developed an attack of bronchitis. She declared herself to be cured.

The subsequent history may not be out of place here, as showing that no relapse has occurred in three years.

March 7th (extract from a letter): "I am so pleased to tell you that I am ever so well—my appetite good and my weight up to 7 st." (an increase of 4 lbs.). "What a change in such a short time, and not all outward and visible! My mother writes that she cannot remember my being ill until I was between 4 and 6 years old, when I had bronchitis and was sufficiently ill to have a doctor. We were then living in the house I described. From then onwards I had attacks of weakness, and when I was twelve the doctor advised my leaving school, which I did." April 4th: "I am still improving, and my weight this morning was 7 st. 6 lbs." June 3rd: "I still feel very well." October 7th: She reported being very well. Weight 7 st. 8 lbs., and, apart from an attack of ptomaine poisoning while abroad, had remained well. 1924: In January there was a mild attack of depression, which cleared up in a few days with suggestion under hypnosis. The weight had fallen to 6 st. 3 lbs. March 11th: "I am now much better and weight about 7 st. again." August 1st: I saw her with a slight attack of rhinitis, which cleared up with a little local treatment. September 26th: An attack of acute gastritis, which responded to medicine and three hypnotic treatments. 1925: Remained well. 1926: Reported that the cure had been successful.

I have selected this case for description at some length, because, firstly, it illustrates certain points common to most cases of repressed memories; secondly, because it proves that a purely mental phenomenon can produce definite physical signs, like a rise of temperature, if such physical conditions occurred at the time of the repression ; thirdly, because it would need a wilfully unnecessary kind of imagination to read any sexual significance into it.

The patient suffered from definite depression, a condition which I have found in nearly all, though not quite all, the cases I have treated. This depression is one of the most serious symptoms to the patient ; and, because of the difficulty of its explanation or even of its description, it is in danger of being neglected. Sometimes it is accompanied by fear ; but, when asked of what he is afraid the patient is unable to say, or gives an evasive answer ; the evasive reply is very suggestive of a repressed memory.

Pain is a common symptom, and appears when some physical injury took place at the time of the repression. Probably in the case described, an attack of pleurisy was induced by the draught from the open window.

A sense of injustice, combined with a marked antipathy towards some person, place or thing, are suggestive symptoms. In this case the patient had an antipathy to women who had any authority over her.

Then there is the interesting reproduction of physical signs. The commonest forms are bruises, definite discoloured patches which appear without apparent cause. Dickens, who had such a wonderful power of observation, has described one upon the lip of Rosa Dartle. I have seen areas of congestion, and even deformities, resulting from successive attacks of inflammation due to these repressed memories. But on the whole physical signs are more marked by their absence than by their presence.

Although sex is sometimes a factor in these cases, it will be found that fear is a more dominating feature ; and if the psycho-analysts had spent as much time and patience in the elucidation of the mystery of fear as they have on the symbols of sex they would have increased the benefit to humanity an hundredfold.

